# Validation of a data-driven multicomponent T_2_ analysis for quantifying myelin content in the cuprizone mouse model of multiple sclerosis

**DOI:** 10.1371/journal.pone.0323614

**Published:** 2025-05-21

**Authors:** Noam Omer, Ella Wilczynski, Sharon Zlotzover, Coral Helft, Tamar Blumenfeld-Katzir, Noam Ben-Eliezer

**Affiliations:** 1 Department of Biomedical Engineering, Tel Aviv University, Tel Aviv, Israel; 2 Sagol School of Neuroscience, Tel Aviv University, Tel Aviv, Israel; 3 Center for Advanced Imaging Innovation and Research (CAI2R), New York University School of Medicine, New York, United States of America; Public Library of Science, UNITED KINGDOM OF GREAT BRITAIN AND NORTHERN IRELAND

## Abstract

**Background:**

Myelin quantification is essential for understanding a wide range of neurodegenerative pathologies. Voxel-wise multicomponent T_2_ (**mcT**_**2**_) analysis is the common approach for this purpose, yet no gold standard technique exist that can overcome the ambiguity of fitting several T_2_ components to a single-voxel signal. This challenge is further exacerbated in preclinical scan settings due to the addition of spurious diffusion encoding, resulting from the use of imaging gradients that are at least an order of magnitude larger than on typical clinical scanners.

**Purpose:**

Assess the utility of a new data-driven approach for mcT_2_ analysis, which utilizes information from the entire tissue to analyze the signal from each voxel in healthy and demyelinated tissues. Specifically, this algorithm uses statistical analysis of the entire anatomy to identify tissue-specific multi-T_2_ signal combinations, and then uses these as basis-functions for voxel-wise mcT_2_ fitting.

**Methods:**

Data-driven mcT_2_ analysis was performed on N = 7 cuprizone mice and N = 7 healthy mice. Myelin water fraction (**MWF**) values at six brain regions were evaluated. Correlation with reference immunohistochemical (**IHC**) staining for myelin basic protein was done in the corpus callosum. To demonstrate the added value of the data-driven approach the analysis was performed twice – with and without the data-driven preprocessing step.

**Results:**

Strong agreement was obtained between data-driven MWF values and histology. Applying the data-driven analysis prior to the voxel-wise fitting improved the mapping accuracy vs. non data-driven analysis, producing statistically significant separation between the two mice groups, good groupwise linear correlation with histology (cuprizone: R² = 0.64, *p* < 0.05, control: R^2 ^= 0.61, *p* < 0.05), and addressed the inherent ambiguity, characterizing conventional mcT_2_ fitting.

**Conclusion:**

The proposed data-driven algorithm provides a reliable tool for mapping myelin content on preclinical scanners, allowing precise classification between healthy and demyelinated tissues in cuprizone mouse model of multiple sclerosis.

## Introduction

Myelin is linked to almost every neurological function through its involvement in neuronal processing and connectivity. Characterizing the brain myeloarchitecture is therefore essential for understanding both neurodevelopmental processes and aging [1–3]. Myelin is also involved in a wide range of neurological and mental disorders including multiple sclerosis [4,5], Alzheimer’s disease [6], schizophrenia [7], and dementia. Myelin degeneration is also known to occur in areas of white matter (**WM**) hyperintensities (regions that appear hyperintense on T_2_-weighted MRI scans) and dirty WM – two pre-symptomatic abnormalities that, in many cases, precede the onset of diseases [8,9]. Despite its key neurological functions, quantification of myelin in vivo is still highly challenging with no gold standard approach.

Cuprizone-induced demyelination is an established mice model of myelin damage, commonly used in preclinical studies of multiple sclerosis and other myelodegenerative diseases [10–12]. In this model, demyelination is induced by adding the copper chelator cuprizone to the animal’s diet, leading to selective oligodendrocyte apoptosis, followed by extensive demyelination in WM regions, and particularly in the corpus callosum (**CC**) [13]. The cuprizone model is thus a practical choice for evaluating myelin mapping techniques as it induces controlled demyelination without the formation of focal lesions [14,15].

Myelin content can be probed using several MRI contrasts [16–19]. These include magnetization transfer (**MT**) [20–22], molecular diffusion [23–25], spin-lattice relaxation time (**T**_**1**_) [26–28], spin-spin relaxation time (**T**_**2**_) [29,30], magnetic susceptibility [31–34], and dual-contrast approaches which combine T_1_- and T_2_-weighting [35–39]. Quantitative MRI (**qMRI**) approaches are particularly beneficial in this context as, apart from providing a quantitative measure of myelin content, they improve MRI’s sensitivity to tissue changes [40]. Several techniques have been developed based on these contrasts and used for studying the cuprizone model in mice [41–43]. Among these, multicomponent T_2_ (**mcT**_**2**_) relaxometry is the most effective and popular approach [29,44–51], although its postprocessing is highly ill-posed and lacks reliable validation. Another significant challenge in quantifying myelin arises in preclinical scanners, widely used for drug development and the study of diseases pathophysiology. These high-resolution MRI scanners employ stronger magnetic field gradients, causing spurious signal decay due to molecular diffusion, which, in turn, lead to underestimation of T_2_ values and biased estimation of myelin content.

mcT_2_ mapping of myelin is based on measuring the signal originating from water that reside between myelin sheaths [29,47] and estimating its relative fraction, i.e., the myelin water fraction (**MWF**) [52]. This is done by separating multi-echo spin-echo (**MESE**) MRI signals into a distribution of T_2_ values, i.e., generating a T_2_ spectrum per voxel [30]. In the case of myelinated WM tissues, the MESE signals are typically considered to be a weighted sum of three sub-voxel compartments: intra-cellular, extra-cellular, and the water that reside between myelin sheaths [53]. The MWF is then calculated as the relative energy of the short T_2_ component (e.g., between 10–40 ms), while the intra/ extra-cellular water pools are characterized by longer relaxation times in the range of 40–200 ms and termed *geometric mean* of T_2_ values [52,54]. Traditional voxel-wise mcT_2_ fitting procedures employ a regularized non-negative least squares (**NNLS**) fitting to deconvolve the signal at a given voxel into a weighted combination of single T_2_ components [55,56]:


$$S_{voxel}=\sum_{n=1}^{N_{T_{2}}}w_{n}S^{T_{2,n}}\;s.t.\;\sum w_{n}=1$$ (1)

where $N_{T_{2}}$ is the number of single-T_2_ components $S^{T_{2,n}}$, and $w_{n}$ are their relative fractions. Other multicomponent approaches exist which employ multi gradient-echo acquisition schemes like GRASE [57], magnetic resonance fingerprinting [58,59] and driven equilibrium single pulse observation (mcDESPOT) [60,61]. These approaches offer faster data collection, albeit, at a tradeoff of reduced T_2_ encoding efficiency compared to spin-echo acquisitions [62,63]. Notwithstanding the utility of existing techniques, the accuracy of voxel-wise mcT_2_ approaches is limited, mainly because the SNR in a given voxel is insufficient to overcome the large ambiguity that emerges when fitting multiple unknown parameters to a single signal. This leads to low reproducibility across different techniques [62] and high sensitivity to noise, even in numerical simulations which are rich in SNR [51,64].

In this study we employed a recent data-driven mcT_2_ algorithm [65] to investigate demyelination in a cuprizone mouse model of MS under preclinical scan settings. The data-driven algorithm includes a pre-processing step, which harnesses concepts from probability theory in order to identify a specific set of multi component signatures, termed ‘motifs’, which best describe the tissue under investigation. These mcT2 motifs are then used to deconvolve the signal from each voxel, thereby enhancing the algorithm’s stability and accuracy in generating myelin water fraction (MWF) maps, compared to non data-driven approaches. [65,66]. This study presents application of the algorithm on a group of cuprizone-fed mice and a group of healthy mice, followed by comparing MWF values in the CC of each mouse to reference values obtained using immunohistochemistry (**IHC**). The added value of the data-driven preprocessing approach is further demonstrated by comparing MWF maps produced using rNNLS fitting with and without a data-driven pre-processing step.

## Methods

### Animal model

Fourteen six-weeks old mice (C57BL⁄6) were divided into two groups: one group (N = 7) underwent a 6-week cuprizone-rich diet, administered in powder form, mixed with standard chow to achieve a final concentration of 0.3% [10,67]. The second, control group, consisted of age-matched healthy mice (N = 7) which were put on a normal diet. Following the six-weeks diet all mice underwent MRI scans, followed by euthanization and histological analysis. The study was carried out in accordance with the ethical guidelines of the animal care unit of the faculty of medicine, Tel Aviv University (approval No. M-01-17-095).

### MRI scans

Scans were performed on a 7 T Bruker BioSpec scanner (Bruker Biospin, Germany), using single-channel transmit and four-channel receive head coil. Imaging used a MESE protocol with the following parameters: TR/TE = 3000/5.5 ms, echo-spacing = 5.5 ms, FOV = 20 × 20 mm^2^, matrix size = 128 × 128, N_averages _= 2, echo train length (ETL) = 20, slice thickness = 0.8 mm, N_Slices _= 2, slice gap = 100%, acquisition bandwidth = 390 Hz/Px. Total scan time was 12:48 minutes per mouse. During MRI scans mice were anesthetized with isoflurane gas (1–2% in O_2_), their body temperature maintained at 37°C using a water circulation system, and their breathing probed using an abdominal pressure pad.

### Histology-based estimation of myelin content

Following the MRI scans, mice were anesthetized with ketamine and xylazine and perfused with 4% paraformaldehyde in a phosphate buffer saline. Subsequently, the brains were extracted and fixed overnight in the same fixative solution (at 4°C), and then transferred into a 1% paraformaldehyde solution. Next, brain samples were embedded in paraffin blocks, sliced transversely at a thickness of ~ 4 µm, and situated on separate glass slides.

The tissue sections were deparaffinized using xylene, followed by rehydration through a graded ethanol series (100%, 95%, 70%, and 50%) and finally rinsed in distilled water. For antigen retrieval, the sections were treated using the Leica Bond-max system (Leica Biosystems, UK) with epitope-retrieval solution at pH 6.0 for 5 minutes. Blocking was done with 5% normal mouse serum in PBS for 1 hour at room temperature to minimize non-specific binding.

Finally, the sections were incubated in anti-myelin basic protein (**MBP**) antibody (1:100, Proteintech USA) for 30 minutes for staining. After this, the secondary antibody, goat anti-rabbit IgG conjugated to horseradish peroxidase (HRP), was applied, and the Leica Refine-HRP kit (Leica Biosystems, UK) was used for detection. DAB (3,3’-diaminobenzidine) was used as the chromogen, followed by counterstaining with hematoxylin. The sections were then photographed using an Olympus BX60 microscope (Olympus, Japan) at two magnification factors, x1.25 and x10. The x10 images were focused on the medial corpus callosum (**MCC**) – a highly myelinated brain region, followed by using these images as a qualitative ground truth estimation of myelin content.

Quantification of the myelin content from the histological x10 images was performed in two stages. First, images were converted to HSV color space, and pixels with hue below 24 (out of a theoretical hue range of 0–255) were filtered out using the *ImageJ* color transformation feature which filters out specific colors and enhances the contrast and clarity of the structures of interest [68]. This threshold was selected based on the visual examination of all histological images by a neuroscientist to accurately capture the myelin staining. This step was essential to retain the MBP pixels (brown hue) and to filter out the cells nuclei and background color (blue and gray hues respectively). In the second stage, manual segmentations of the MCC were performed for each mouse. Pixel values in this region were then normalized to a range of 0 and 100, indicating the relative amount of MBP similar to the MWF values calculated using MRI. The reference value for this normalization was selected based on the maximal hue value in the histological slices across all mice.

### MRI-based data-driven mapping of myelin water fraction (MWF)

Prior to the mcT_2_ analysis all MESE data was corrected to remove diffusion-related bias of the measured signals using the algorithm described in [69]. This bias is specific to high-field or preclinical scanners due to their use of high-power imaging gradients, which cause spurious diffusion-encoding, leading to underestimation of T_2_ values. mcT_2_ analysis was then performed on the diffusion-corrected signals using a regularized NNLS (**rNNLS**) algorithm [55,29], in the slice that was anatomically closest to the location of the histological slice. Fitting was done once using the data-driven preprocessing step [65], and once without it. In the conventional approach, MESE data is deconvolved into a series of exponentially decaying signals, each originating from a different sub-voxel compartment, associated with a unique T_2_ value:


$$S_{voxel}=\sum_{n=1}^{N_{T_{2}}}w_{n}\exp\left(-TE/T_{2,n}\right)$$ (2)

Here, $T_{2,n}$ is the transverse relaxation time constant (**T**_**2**_) of each sub-voxel compartment, and TE is the protocol’s echo-time. rNNLS fitting is used to solve for the unknown values $T_{2,n}$ and $w_{n}$ by minimizing the following cost function:


$$\underset{w}{argmin\;\Phi }=\frac{1}{2}{\left\|EW\;-S_{voxel}\right\|}_{2}^{2}+\lambda_{Tik}{\left\|W\right\|}_{2}^{2}\;s.t.\;w_{n}\geq 0,\;\sum w_{n}=1$$ (3)

Here $E\in {\mathbb{R}}^{ETL\;\times \;N_{T2}}$ is a dictionary of logarithmically spaced single-T_2_ signals, generated using the EMC algorithm [70,71], ranging typically between TE/2 and TExETLx2 with ETL denoting the echo train length, $W\in {\mathbb{R}}^{N_{T2}\;\times \;1}$ is an unknown weights vector representing the relative fractions of each element in ***E***, and $\lambda_{Tik}>0$ is the weight of a Tikhonov regularization term [30]. Implementation of the rNNLS algorithm was done in-house using MATLAB’s quadratic programing toolbox (quadprog).

Solving the problem in Eq. 3 is a highly ill-posed process, even when employing regularization, due to the high ambiguity of fitting 5 unknown parameter to a single signal. To overcome this, the data-driven approach replaces the dictionary ***E*** of simulated single-T_2_ signals with a new dictionary 𝔼 consisting of a set of mcT_2_ motifs (i.e., multicomponent combinations of single-T_2_ signals), which best describes the brain segment being analyzed. In its basis, this algorithm includes several key step (***i***) computing a dictionary of theoretical of mcT_2_ motifs, each consisting of a different combination of single-T_2_ signals (millions of elements); (***ii***) calculating the correlation between each element in the mcT_2_ dictionary and the entire set of voxels in the assayed brain region; (***iii***) scoring all mcT_2_ dictionary elements (motifs) according to how well they match the entire anatomy; (***iv***) applying entropy regularizations so as to favor sparser motifs; (***v***) choosing a set of ℒ pseudo-orthogonal mcT_2_ motifs with the highest score. Full description of the algorithm procedures can be found in [65]. To compensate inhomogeneities in the transmit field (B_1_^+^) (which significantly affect MESE decay curves and can lead to inaccurate myelin estimation), a recent extension of data-driven algorithm was utilized. This extension integrates B_1_^+^ into the fitting process, thereby correcting for its inhomogeneities [66].

Following the selection of the final set of ℒ mcT_2_ motifs, it was possible to model the signal from each voxel as a linear combination of mcT_2_ motifs rather than a set of theoretical single-T_2_ decay curves:


$$\begin{array}{c} S_{voxel}=\sum {\mathrm{\backslash nolimits}}_{n=1}^{ℒ}{𝕎}_{n}S_{n}^{mcT_{2}} \end{array}$$ (4)

where ${𝕨}_{n}$ is the weight of each mcT_2_ motif $S_{n}^{mcT_{2}}$. The optimization problem in Eq. 3 was thus adjusted to use the new signal model, yielding:


$$\underset{𝕎}{argmin\;\Phi }=\frac{1}{2}{\left\|EW-S_{voxel}\right\|}_{2}^{2}+\lambda_{Tik}{\left\|𝕎\right\|}_{2}^{2}\;s.t.\;{𝕎}_{n}\geq 0,\;\sum {𝕎}_{n}=1ℵ$$ (5)

where $E\in {\mathbb{R}}^{ETL\;\times \;ℒ}$ is the set of ℒ mcT_2_ motifs and $𝕎{\in R}^{L\;\times \;1}$ is the set of unknown weights of each mcT_2_ motif in E.

The current study used the following settings for the rNNLS optimization, for both data-driven and conventional non data-driven processing: the original dictionary E contained 200 single-T_2_ values, logarithmically spaced between 1...800 ms, Tikhonov regularization λ_Tikh_ = 0.001 and L_1_ regularization λ_L1_ = 0.01. For the data-driven dictionary (𝔼) mcT_2_ motifs were generated from combinations of single, pairs, and triplet single-T_2_ decay curves, chosen from the same 200 single-T_2_ signals, and relative fractions ${𝕨}_{n}$ at intervals of 0.05, and short T_2_ fraction limit of 30%. Selection of the set of tissue-specific motifs used an entropy regularization of λ_En_ = 0.001, regularization factor for B_1_^+^ correction of μ = 1, and similarity parameter of δ_MV_ = 0.01. Full description of the mcT_2_ algorithm and its parameters can be found in [66].

**Fig 1:**
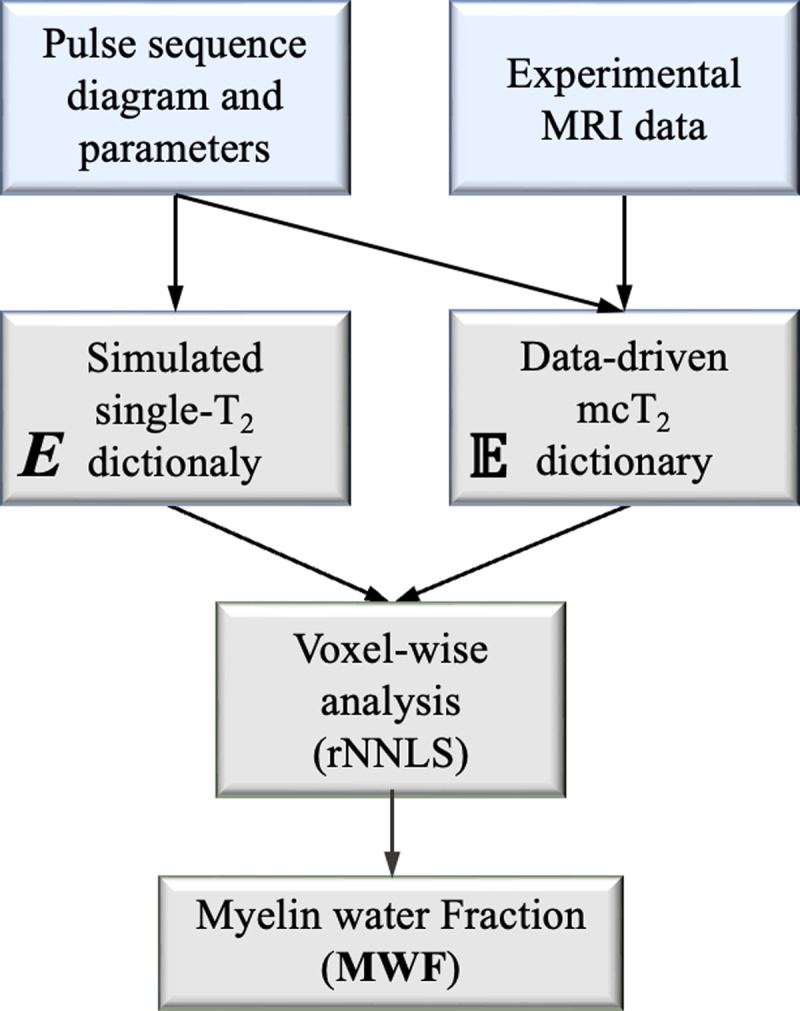
Flowchart of the data-driven vs. the conventional approach. While both approaches extract the pulse sequence diagram and scan parameters in order to generate experimentally-realistic decay curves and maintain consistency across scanners and scan settings [70,71], the data-driven approach integrates actual scan data into a preprocessing stage that produces a tissue-specific dictionary of mcT_2_ motifs, □. The conventional approach, in contrast, relies on a simulated dictionary of single-T_2_ decay curves, E, which does not consider the data itself. Both approaches then apply the same regularized nonnegative least square (rNNLS) fitting algorithm to locally deconvolve the signal in each voxel.

MWF values were calculated from the reconstructed spectra at each voxel by summing the area under the peak in the short T_2_ range (0–40 ms) [29]. As noted in Eq. (5) the sum of each weights vector is equal to 1, meaning that this area directly reflected the MWF value. Fig 1 schematically illustrates the data-driven pipeline alongside the conventional approach. We emphasize that the change introduced by the data-driven approach is the use of an mcT_2_ dictionary 𝔼, which is learned from the data, whereas the conventional (non data-driven) approach employs a theoretical dictionary of single-T_2_ decay curves. Both approaches, however, utilize the same fitting algorithm to the reconstruct the MWF within each voxel.

### Statistical analysis

Mean and standard deviation (**SD**) of normalized, histology-derived, myelin content were extracted for the MCC of each mouse. The statistical difference between the cuprizone and control groups was then assessed using a two-tailed *t*-test, with degrees of freedom (df) calculated based on the sample sizes of each group. A significance level of α = 0.05 was used for hypothesis testing. Since the test was two-tailed, the significance level for each tail was 0.025. The critical t-value for df = 12 at α = 0.05 was $t_{critical}\approx$ 2.179. Mean and standard deviation (**SD**) of MRI-derived MWF values were calculated for six two-dimensional regions of interest (**ROIs**), according to mouse brain atlas [72]. Segmentation of ROIs was performed by a trained neuroscientist on the MESE images (4^th^ echo). ROIs included the cortex, MCC, external corpus callosum (**ECC**), thalamus, hippocampus (**HIPP**), and striatum. To mitigate partial volume effects ROIs were subjected to a two-pixels erosion, and a weak outlier removal was done by excluding pixels with MWF values exceeding the mean by more than ±3 SD. To evaluate the impact of the cuprizone diet on MWF values, the statistical differences between MWF from the cuprizone and control groups was compared for each ROI, using a two-tailed *t*-test of the mean. The degrees of freedom (df) for *t*hese tests were based on the sample sizes of each group, and strict post-hoc Bonferroni correction was applied to account for multiple comparisons. This correction adjusts the p-value threshold by dividing the significance level by the number of comparisons (in our case, 0.05/6 = 0.008). Finally, the Pearson linear correlation (R² and *p*-value) was calculated between histology-derived myelin content and MRI-derived MWF values in the MCC. This process was repeated twice: once using the data-driven approach and once using the conventional non data-driven approach.

## Results

### Histology-based estimation of myelin content

**Fig 2:**
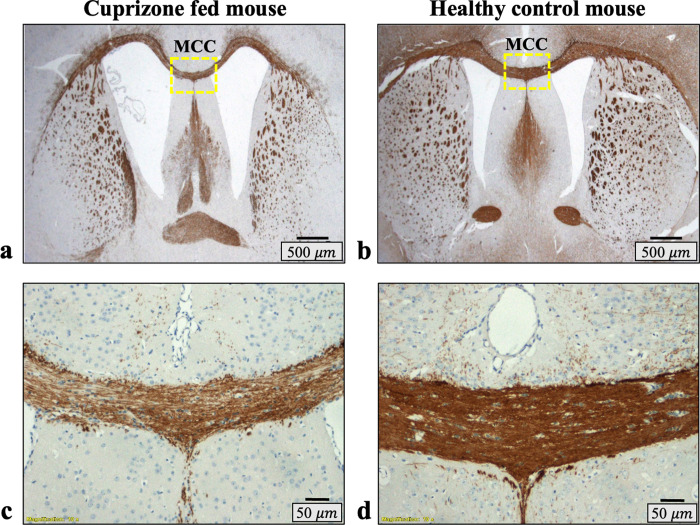
Representative cross-section histology images stained with myelin-basic-protein, where darker brown hue denotes higher level of MBP staining. **(a-b)** microscopy scans of a histological slide containing the entire corpus callosum (**CC**). **(c-d)** Zoomed images of the slices in **(a-b)**, focusing on the medical part of the corpus callosum (**MCC**) used to evaluate the agreement with MRI-derived MWF values. Demyelination is clearly seen along the CC of the cuprizone-fed mouse (a,c) vs. healthy control mouse **(b,d)**. Additional slides showing varying levels of demyelination are presented in Supplementary S1 Fig.

**Fig 3:**
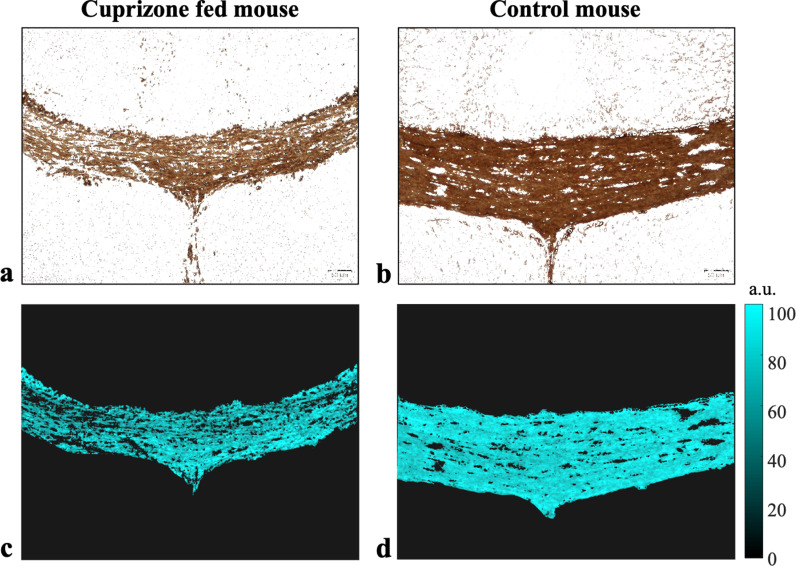
Example of histological analysis of myelin content at the medial corpus callosum (MCC). Myelin-basic-protein (**MBP**) channel from immunohistochemical (IHC) staining is shown at the MCC for **(a)** Cuprizone-fed mouse presenting thin and porous staining, and **(b)** a healthy control mouse. **(c-d)** Matching masks.

Representative cross-sections of MBP-stained brain slices (x1.25) are shown in Fig 2a-b for a control and a cuprizone-fed mouse. Visual examination of the histological slices from the control mice showed normal brain morphology with stronger (i.e., darker brown hue) and denser MBP staining along the CC compared to the cuprizone-fed mice. Histology images of the cuprizone-fed mice showed variable disease severity across the group. Mildly affected mice exhibited lower staining intensity (i.e., lighter brown hue) and sparser staining compared to controls, while the more severe cases exhibited much lighter brown hue alongside morphological alterations such as dilated ventricles and thinning of the CC. Magnified views (x10) of healthy and cuprizone MCC that were used for histological analysis of myelin content are shown in Fig 2c-d, highlighting the higher myelin content in the control mouse. Quantitative analysis of histology images of the MCC and its surrounding was done by converting them into monochromatic images, where the color intensity correlates with MBP staining (representative example in Fig 3a-b). Manually segmented masks of the MCC are shown in Fig 3c-d, normalized to 0…100 according to the maximal hue value of 221 across all mice. Statistical analysis of hue strength (corresponding to myelin content), averaged across the MCC for each mouse, produced statistically significant difference between control and cuprizone-fed groups (*p* < 0.001), exhibiting non-overlapping ranges with mean ± SD myelin content of 31.6 ± 3.1 for the cuprizone group and 58.1 ± 5.0 for the control mice.

The two-sample t-test was performed to compare the normalized histology-derived myelin content in the MCC of the two groups. Analysis yielded a $t_{statistic}$ of 6.83. Since this value is significantly larger than the critical $t_{critical}\approx$ 2.179 (calculated above), the difference between the groups is statistically significant at the 0.05 significance level. The coefficient of variation (CV) of myelin content within each ROI, averaged across mice, was 9% in the control group and 10% in the cuprizone group, indicating a relatively low level of variability in both groups with no statistically significant difference observed between the two groups.

### MRI-based mapping of myelin content

**Fig 4:**
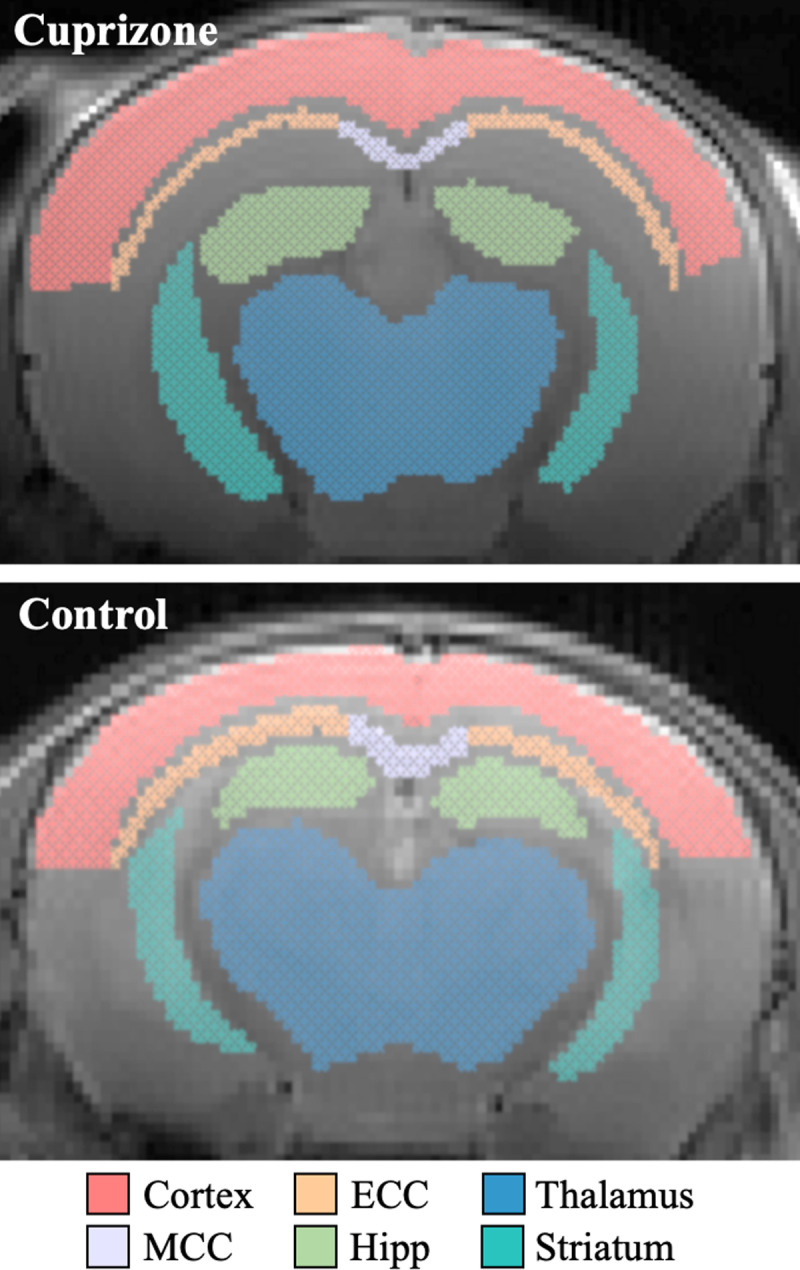
Representative segmentations of brain ROIs, used for analyzing MWF values. Six brain ROIs are shown for **(a)** cuprizone-fed and **(b)** control mice. ROIs include: cortex, medial corpus callosum (MCC), external corpus callosum (ECC), hippocampus (HIPP), thalamus, and striatum. Segmentation was performed manually according to the Allen Mouse Brain Atlas (mouse P56, Coronal images: 68-72) [73].

**Fig 5:**
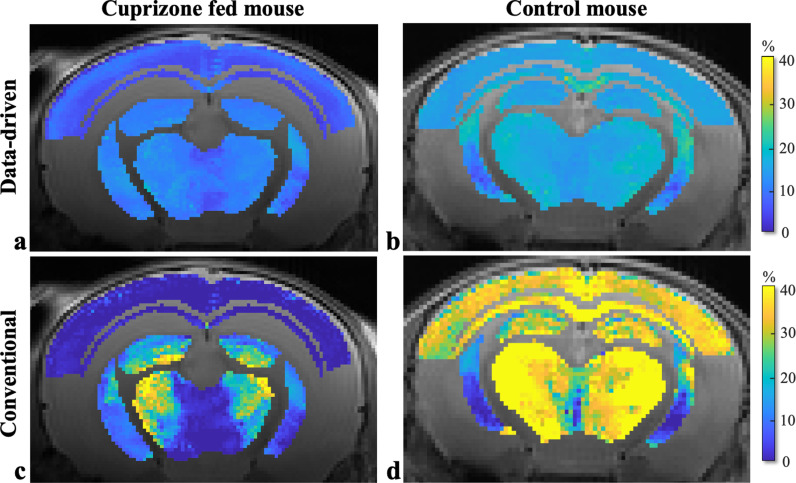
MRI-derived MWF maps for six brain regions in cuprizone and control mice using data-driven and conventional approaches. Masked ROIs are overlaid on a T_2_-weighted MESE image (4^th^ echo time). **(a-b)** Maps generated using the data-driven mcT_2_ approach. **(c-d)** Maps generated using the conventional approach (i.e., without the additional data-driven preprocessing step). Additional MWF maps can be found in Supplementary S2 Fig.

mcT_2_ analysis was applied to the six ROIs illustrated in Fig 4, using both the data-driven and conventional (non data-driven) approach. Analysis generated MRI-derived MWF maps for each ROI within each mouse brain. Fig 5 presents examples of two such MWF maps, produced using both approaches for cuprizone-fed and control mice (Fig 5a,c and Fig 5b,d, respectively), overlaid on T_2_-weighted images (4th echo, MESE protocol). While both methods show lower MWF values in the cuprizone-fed mice, the conventional approach produces high and non-physiologic variations in MWF values, while also overestimating MWF values in the healthy control mice. Whole brain MWF maps are shown in Supplementary S2 Fig.

**Fig 6:**
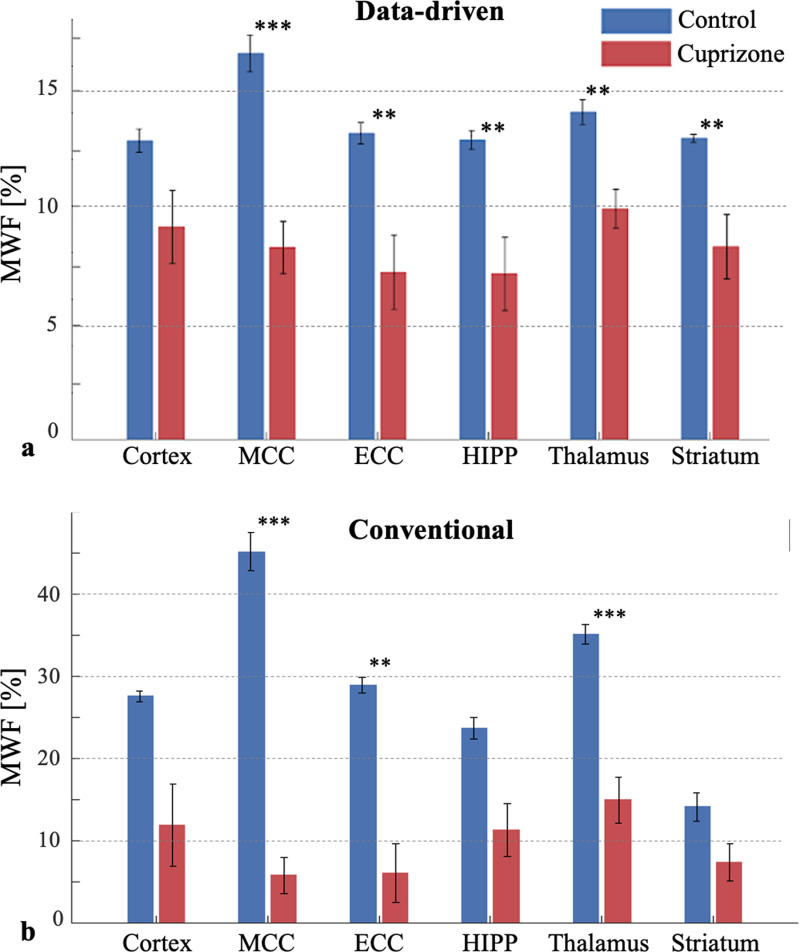
Mean and SD of MRI-derived myelin water fraction (MWF) values calculated using (a) data-driven approach, and (b) the conventional approach. Both approaches produced lower MWF values in the cuprizone-fed mice group (red) compared to the control group (blue) across all brain ROIs. Error bars represent the standard deviation in MWF values. Bonferroni corrected *p*-values, are indicated by asterisks for each region *p < 0.05, **p < 0.01, ***p < 0.001).

Groupwise analysis of MRI-derived MWF values is presented in Fig 6 revealing that both approaches produce lower mean values and higher SDs (calculated across mice) for cuprizone-fed mice compared to the control group across all brain ROIs. Before applying the Bonferroni correction, we observed statistically significant differences in the mean MWF estimates across all regions with both methods. After the correction, the difference in the Cortex (p = 0.0204) became not significant using either approach. All five remaining ROIs, however showed statistically significant differences in the data-driven approach:

MCC (***p < 0.001), ECC (**p < 0.01), HIPP (**p < 0.01), thalamus (***p < 0.001), and striatum (**p < 0.01), while the conventional approach only showed significant differences in the MCC (***p < 0.001), ECC (***p < 0.001), and Thalamus (***p < 0.001). The highest statistical difference was observed in the CC, which aligns with previous literature indicating that the cuprizone model predominantly affects this region.

The percentage of excluded outlier MWF values across the ROIs was 1.68% for control and 1.46% for cuprizone using the data-driven approach, and 1.61% for control and 1.55% for cuprizone using the conventional approach. Additionally, the analysis highlights the overestimation of the conventional approach. According to Table 1 this observation is particularly notable in the MCC, where the mean MWF obtained with the conventional approach exceeded 40%, leading to ~ x5 times difference between control (45.1%) vs. cuprizone-fed (5.8%) groups, compared to a much more physiological difference of 16.6% for control vs. 8.2% for the cuprizone-fed mice when using the data-driven approach.

**Table 1:** Mean, standard deviation (SD) and coefficient of variation (CV) of MRI-derived myelin water fraction (MWF) in the medial corpus callosum (MCC). Values were obtained using the data-driven mcT_2_ approach and using the conventional approach. Rightmost column contains reference values, derived from histology.

Group	MRI-derived MWF in MCC [%]						Histological analysis in MCC [a.u.]		
	Data-driven			Non data-driven					
	Mean	SD	CV	Mean	SD	CV	Mean	SD	CV
Control	16.6	2.8	17%	45.1	9.7	21%	58.1	5.0	9%
Cuprizone	8.2	1.2	15%	5.8	4.2	72%	31.6	3.1	10%

### Correlation between histology and MRI-based mapping of myelin content

**Fig 7:**
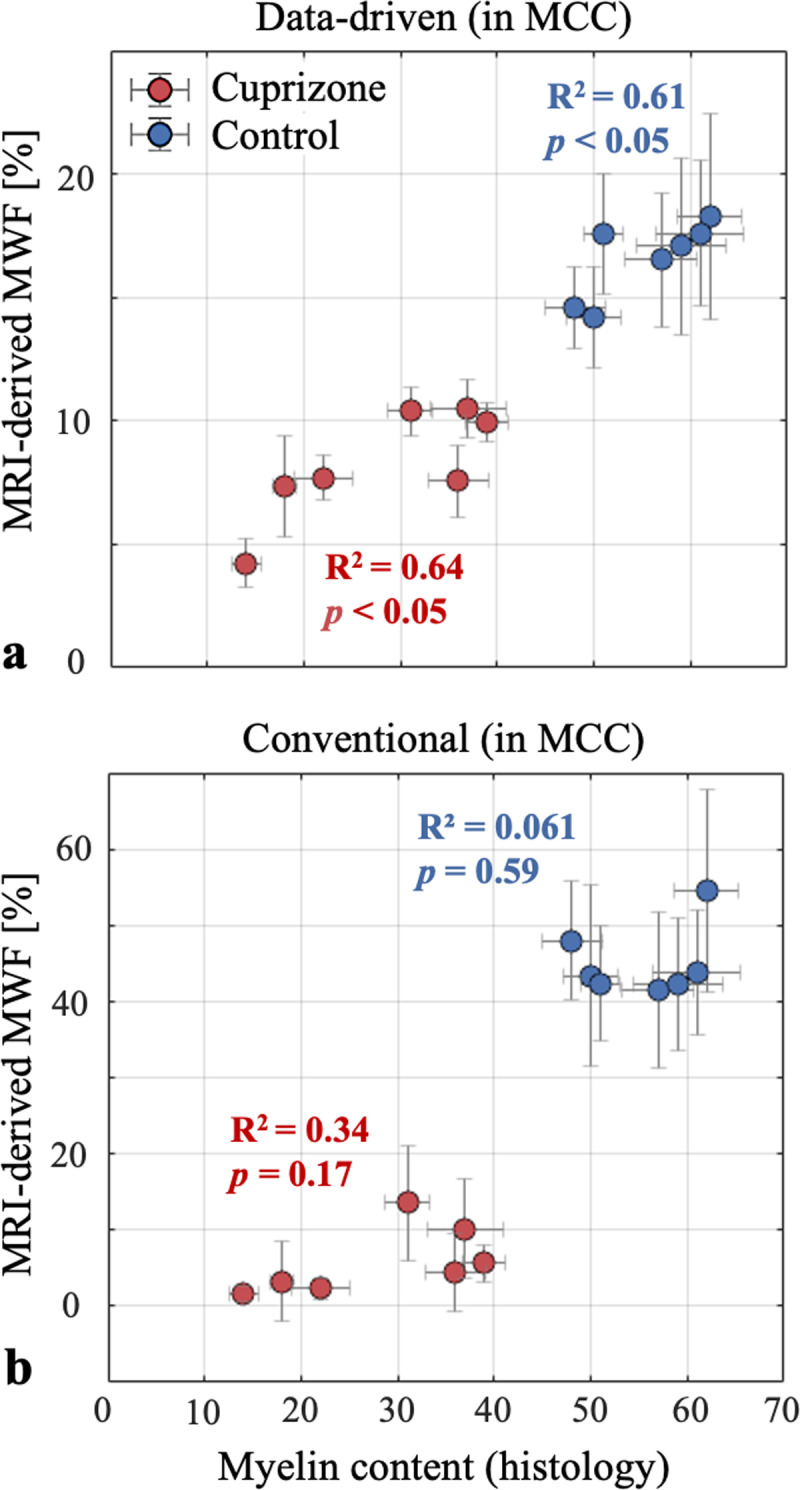
Scatter plot of MRI-derived MWF values for each mouse across the medial corpus callosum (MCC), plotted against corresponding histology-derived myelin content. Cuprizone-fed and control groups are marked with dark red and dark blue circles, respectively. Mean MWF values were calculated using **(a)** the proposed data-driven mcT_2_ approach [65], and **(b)** the conventional approach. Both methods, as well as the histology data, indicate reduced myelin content in the cuprizone-fed group and a distinct separation between the groups (*p* < 0.0001 for both methods). The data-driven approach, however, demonstrates a strong linear correlation with histology-derived values for both the control group (*R*² = 0.61, *p* < 0.05) and the cuprizone group (*R*² = 0.64, *p* < 0.05), while the conventional approach produced no linear correlation with histology-derived values in the control group (*R*² = 0.06, *p* = 0.59) or the cuprizone group (*R*² = 0.34, *p* = 0.17). Error bars on the horizontal and vertical axes represent the standard deviation (see Supplementary S1 Table for specific numerical values).

Table 1 presents MRI-derived MWF values in the MCC for each mouse, alongside the corresponding histology-derived values. The coefficient of variation (CV) of MRI-derived MWF in the MCC was significantly higher when using the conventional approach, particularly in the cuprizone group, compared to histology (see also Supplementary S1 Table). In contrast, the data-driven approach produced CV values comparable to those derived from histology. A scatter plot of MRI-derived MWF values vs. histology-derived values in the shown in Fig 7 for the MCC of each mouse. Error bars represent the standard deviation of MWF values across the MCC. Two distinct clusters emerge when using data-driven analysis (Fig 7a), clearly separating the cuprizone and control groups and having linear correlation with histology-derived values for both the control group (*R²* = 0.61, *p* < 0.05) and the cuprizone group (*R²* = 0.64, *p* < 0.05). A two-tailed *t*-tes*t* comparing MWF values between the cuprizone and control groups produced a statistically significant difference (*p* < 0.001) for both MRI and histology derived values. The added value of the data-driven approach is highlighted in Fig 7b, which shows that the conventional non-data driven approach is able to separate between the cuprizone and control groups, albeit, produces near-zero correlation with histology-derived values, with *R*^*2 *^= 0.06 for the control group (*p *= 0.59), and *R²* = 0.34 for the cuprizone-fed group (*p* = 0.17).

Regions specific values are presented in Table 2. As expected, higher MWF values were produced for the control group, using both data-driven and conventional approaches. The conventional approach, however, overestimated MWF values up to non-physiological of 45.1%. Smaller SD values were observed in the control group in all examined ROIs, indicating more homogeneous distribution of myelin in the healthy tissue.

**Table 2:** Mean and standard deviation (SD) of MRI-derived myelin water fraction (MWF) values calculated using the data-driven mcT_2_ approach and the conventional approach for the two mice groups and across six brain ROIs: cortex, medial corpus callosum (MCC), external corpus callosum (ECC), hippocampus (HIPP), thalamus, and striatum. All relevant data are available at data-driven-mcT2-cuprizone-validation GitHub repository and can be accessed at: https://github.com/NoamBenEliezer/data-driven-mcT2-cuprizone-validation.

	Data driven MWF				Conventional MWF			
	Control		Cuprizone		Control		Cuprizone	
	Mean	SD	Mean	SD	Mean	SD	Mean	SD
Cortex	12.8	1.0	9.1	3.1	27.6	1.3	11.9	10.0
MCC	16.5	1.6	8.2	2.2	45.1	4.6	5.8	4.4
ECC	13.1	0.9	7.2	3.2	28.9	2.0	6.1	7.1
Thalamus	12.7	0.9	7.8	3.1	23.7	2.6	11.3	6.4
Hipp	14.0	1.0	9.9	1.7	35.1	2.3	15.0	5.6
Striatum	12.9	0.3	8.3	2.8	14.1	3.4	7.4	4.5

## Discussion

The presented study assessed the applicability of a new data-driven algorithm for mapping MWF values in a cuprizone mouse model of MS based on mcT_2_ analysis of MESE MRI signals [65]. The algorithm was applied to a group of seven cuprizone-fed mice and seven healthy mice, added with a localized comparison of myelin in the MCC to qualitative estimation done using immunohistochemistry staining for MBP. To evaluate the specific contribution of the data-driven preprocessing step [55,29], we performed the analysis with and without this step, prior to applying voxel-wise rNNLS mcT_2_ fitting. This allowed to directly assess the impact of the data-driven step on the ensuing MWF values. Results show higher agreement between the data-driven MWF and reference values (Fig 7) with higher accuracy and precision compared to the conventional approach.

Most mcT_2_ fitting schemes are based on deconvolving MESE signals into a linear combination of theoretical single-T_2_ signals. The large number of unknowns in a standard three- or even two-component analyses, however, leads to high ambiguity and instability of the calculated MWF values. The data-driven approach proposed in [65,66] considers the signal in each voxel as a linear combination of precalculated mcT_2_ motifs, which are learned from the examined anatomy, rather than based on the myriad theoretical combinations of mcT_2_ motifs (Fig 1). This is done by harnessing concepts from probability theory to identify a small and finite set of mcT_2_ motifs that best represent the tissue, and using these as basis-functions in voxel-wise rNNLS fitting.

The lower MWF values obtained for the cuprizone vs. control mice, indicates the presence of demyelination in all examined ROIs. This result aligns with previous studies which have shown that demyelination in cuprizone-treated mice also occurs in the thalamus [74] and cortex [75]. The higher SD values were observed in the cuprizone-treated mice across in all examined ROIs (Table 2), suggesting that the cuprizone-treated brain is more heterogeneous than the healthy brain, in congruence with previous observations in humans by Shepherd et al [76].

### Added value of data-driven approach

The statistically significant group separation attests to the ability of the data-driven approach to classify healthy and demyelinated tissues in vivo based on MWF values. Moreover, the strong linear correlations observed between MRI-derived MWF and histology-derived myelin content in the MCC of both cuprizone-treated and healthy mice suggest that data-driven MWF values can serve as a reliable biomarker for myelin content (see Fig 7a). The conventional approach, in contrast, showed non-significant correlation (see Fig 7b) and overestimated MWF values in all ROIs of the control group and variable bas in the cuprizone group [77]. The significantly enhanced correlation between MRI- and histology-derived myelin values when using the data-driven approach in the cuprizone group highlights the particular importance of employing the data-driven approach in pathological cases where MWF is low. These cases pose greater challenges for quantifying myelin content, yet are important for tracking disease progression or monitoring treatment efficacy. The generally smaller SD and CV values obtained using the data-driven step (Table 1 and Supplementary S1 Table) cannot be validated due to a lack of a genuine ground truth, yet they suggest that the shift from a large dictionary of theoretical single-T_2_ signals to a small set of mcT_2_ motifs endows the analysis with higher stability (see also Fig 6).

The ability of data-driven mcT_2_ fitting to detect subtle changes in MWF values coincide with previous results for phantom scans, where the technique was shown to detect changes in MWF values even at low fractions of {2%, 4%, …, 10%, 15%}. These subtle changes are difficult to detect with conventional fitting, and can indicate remyelination, mixture of de- and re-myelination, and is also influenced by inflammation, and oligodendrocyte-axon interactions. As such, this technique may provide valuable insights into the efficacy of remyelination therapies. Furthermore, this ability enables identification of subtle demyelination in normal-appearing tissues, as well as localized foci of demyelination in animal models that do not exhibit global demyelination like schizophrenia, depression, and Alzheimer’s disease.

### Cuprizone model of demyelination

Cuprizone fed mice constitute an effective model of demyelination [11], exhibiting prominent and consistent loss of myelin in the CC after a 6 week period of cuprizone diet [25]. Histological analysis, done in this study, agreed with these earlier reports, demonstrating presence of the medial-to-lateral gradient of demyelination, significant reduction in MBP in the MCC, and minor decreases in the cortical and thalamic brain regions. High-resolution histological images of control mice also agreed with literature, showing strong MBP staining throughout the CC [78]. Analysis of the intensity of histology staining produced distinct separation between the two mice groups. These histology-derived values, however, depend on a pipeline of laboratory procedures including, staining quality, and the image-processing parameters, and therefore constitute only a qualitative (rather than quantitative) measure of myelin content.

The similar statistically insignificant CV in both groups suggest that the response to cuprizone-induced demyelination is relatively consistent across mice, and the measured variation in myelin content is not influenced by group membership. This finding may be important for assessing the reliability of the myelin content measurements across animals, ensuring that any observed differences between the groups are primarily due to the treatment and not increased biological variability.

The variability in the extent of demyelination seen in this study across the CC is a known feature of the cuprizone model, supported by several studies. Kipp et al. [79] reports that cuprizone-induced demyelination predominantly affects the caudal portion of the corpus callosum, particularly in the midline regions, while the rostral corpus callosum exhibits more pronounced demyelination in the lateral fibers. Xie et al. [80] demonstrated that demyelination in the CC follows a heterogeneous spatiotemporal pattern, with the splenium exhibiting more extensive medial demyelination and the genu showing greater lateral involvement. Similarly, Schmidt et al. [81] reported that cuprizone-induced demyelination is more pronounced in the midline of the CC and medial parts of the cingulum, while lateral regions of the CC, the lateral cingulum, and the fornix appear more resistant to cuprizone exposure. Additionally, Steelman et al. [82] found that demyelination was limited in the rostral region of the CC but nearly complete in the caudal region, beginning at approximately -0.5 mm from bregma. These findings highlight the regional variability of myelin loss across different callosal subregions and underscore the importance of considering spatial differences when interpreting experimental demyelination models.

The 6-week cuprizone diet significantly upregulates microglia in the tissue, which could potentially influence imaging findings. The data-driven approach employed here utilizes MESE data, which induces magnetization transfer (**MT**) effects due to the extensive use of radiofrequency (RF) pulses. This, in turn, lead to sensitivity of the signals to changes in macromolecular content, especially in the context of microglial proliferation.

The extent of this effect depends on the scan settings and may lead to an underestimation of T_2_ values, due to intra-slice saturation of the macromolecules and inter-slice crosstalk [83]. Moreover, MT can have different effect on each cellular compartment, resulting in biased MWF values. In this study we minimized this effect using slice interleaving and a relatively large slice gap of 100%. Although outside the scope of the current study, this topic should be further investigated to assess whether, and to what extent, scan specific MT effects and cuprizone-derived microglial proliferation may influence MWF values.

### Study limitations

Apart from the CC, cuprizone is also known to affect other WM structures such as, cingulum peduncles, and optic tracts [25]. Histological analysis of myelin content in these regions would have strengthen the study conclusions, while future investigation should cover additional regions and slice orientations that will capture additional WM structures.

This study compared *qualitative* post-mortem histological analysis to *quantitative* MRI-derived values acquired in vivo. Perfect agreement is thus not necessarily expected as values might change when excising the tissue, and during IHC preparation stages which might cause mechanical damage, and have unreproducible effect of the chemical fixatives. Another important aspect is the limited spatial resolution of MRI compared to the histological analysis which used around x100 thinner slices (several microns) and x800 higher in-plane resolutions. To overcome this difference, we performed an ROI-based rather than voxel-based comparison on the MCC – a highly myelinated brain region. This approach limits the comparison as it does not allow to perform the comparison at a voxel level. It is therefore hard to determine if the smaller SD values that that were obtained using the data-driven step reflect the true tissue microstructure, or to infer whether this result can also serve as an indication of the heterogeneity of MWF values at the millimetric mesoscale.

While MBP is a well-established marker for myelin, it does not fully capture the broader changes in myelin and lipid content that may occur during cuprizone treatment. Luxol Fast Blue (**LFB**) staining could provide complementary insights by highlighting neutral lipids and offering a more detailed assessment of myelin degradation in the corpus callosum. However, in this proof-of-concept study, histology served primarily as a reference for correlating with in vivo MWF mapping rather than investigating specific biological mechanisms. Future studies may benefit from incorporating LFB staining for a more comprehensive histological evaluation of the cuprizone model.

The use of cuprizone powder may have contributed to the relatively low extent of demyelination observed. Toomey et al. [84] found that cuprizone pellets were more effective at inducing demyelination, astrogliosis, microglial activation, DNA damage, and oligodendrocyte depletion, especially in the rostral and caudal CC. Future experiments using cuprizone pellets may enhance the extent of demyelinating by the study endpoint.

Additional limitations of this study include the relatively small sample size, variability in the location of histological sampling between mice, and the use of a single time point for assessing pathology. Incorporating postmortem human tissue with immunohistochemical analysis could further validate the method and increase its relevance to human MS. Future research should include data from multiple disease stages and a larger cohort to enhance robustness. Expanding the study to earlier time points would also allow assessing the sensitivity of the data-driven approach to subtle myelin changes and its potential for early detection of pathology. This is particularly important given the distinct biological mechanisms at different time points, e.g., Joost et al. (2021) which reported vacuolization in the medial CC as early as 1–3 weeks following cuprizone exposure.

## Conclusions

The current study demonstrates the utility of a new data-driven algorithm for mcT_2_ analysis and as a reliable tool for quantitative imaging of demyelination. The results indicate that the proposed method can classify healthy and pathological tissues under preclinical settings and provide an accurate and quantitative biomarker for myelin content in vivo.

## Supporting information

S1 Fig Histology images of three cuprizone-fed mice showcasing the variability in the extent of demyelination following cuprizone diet. Images were stained with myelin-basic-protein (**MBP**), where darker brown hue denotes higher level of MBP staining. **(a-c)** microscopy scans of a histological slide containing the entire corpus callosum (**CC**). **(d-f)** Zoomed images, focusing on the medical part of the corpus callosum (**MCC**) which was used to evaluate the agreement with MRI-derived MWF values. Images are sorted by demyelination severity, with the left column showing mild demyelination, the center column showing moderate demyelination, and the right column showing strong demyelination.
(TIFF)

S2 Fig MRI-derived myelin water fraction (MWF) maps produced by the data-driven and conventional (non data-driven) approaches in a cuprizone-fed mouse and a healthy control mouse. **(a-b)** Maps generated using the data-driven mcT_2_ approach. **(c-d)** Maps generated using the conventional approach. Maps are overlaid on a T_2_-weighted MESE image (4^th^ echo time). Both methods show lower MWF values for the cuprizone mouse, however, the conventional approach overestimates MWF in the healthy control mouse and in the thalamus.
(TIFF)

S1 Table MRI-derived myelin water fraction (MWF) values calculated in medial corpus callosum (MCC) versus ground truth values from histology. Mean, standard deviation (SD) and coefficient of variation (CV) are shown for the data-driven mcT_2_ approach and using the conventional approach for each mouse. Rightmost columns present the corresponding histology-based values of myelin content.
(TIFF)
