# Supplementary figures and images for: Validation of a data-driven multicomponent T2 analysis for quantifying myelin content in the cuprizone mouse model of multiple sclerosis
Source: PLoS One. 2025 May 21;20(5):e0323614. doi: 10.1371/journal.pone.0323614 (PMC12094733; doi:10.1371/journal.pone.0323614)

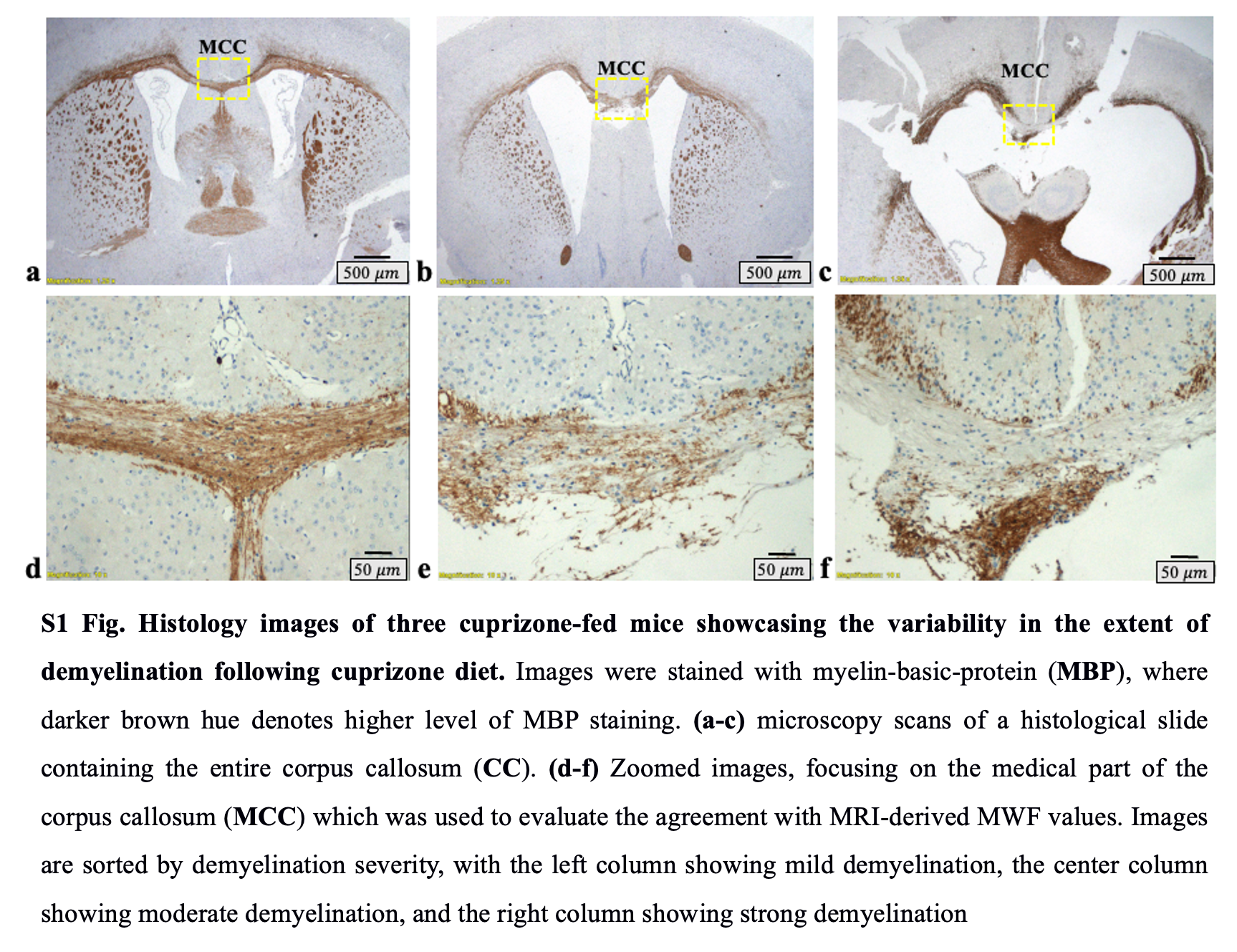

Supplement: S1 Fig — Images were stained with myelin-basic-protein (MBP), where darker brown hue denotes higher level of MBP staining. (a-c) microscopy scans of a histological slide containing the entire corpus callosum (CC). (d-f) Zoomed images, focusing on the medical part of the corpus callosum (MCC) which was used to evaluate the agreement with MRI-derived MWF values. Images are sorted by demyelination severity, with the left column showing mild demyelination, the center column showing moderate demyelination, and the right column showing strong demyelination. (TIFF) [file pone.0323614.s001.tiff]

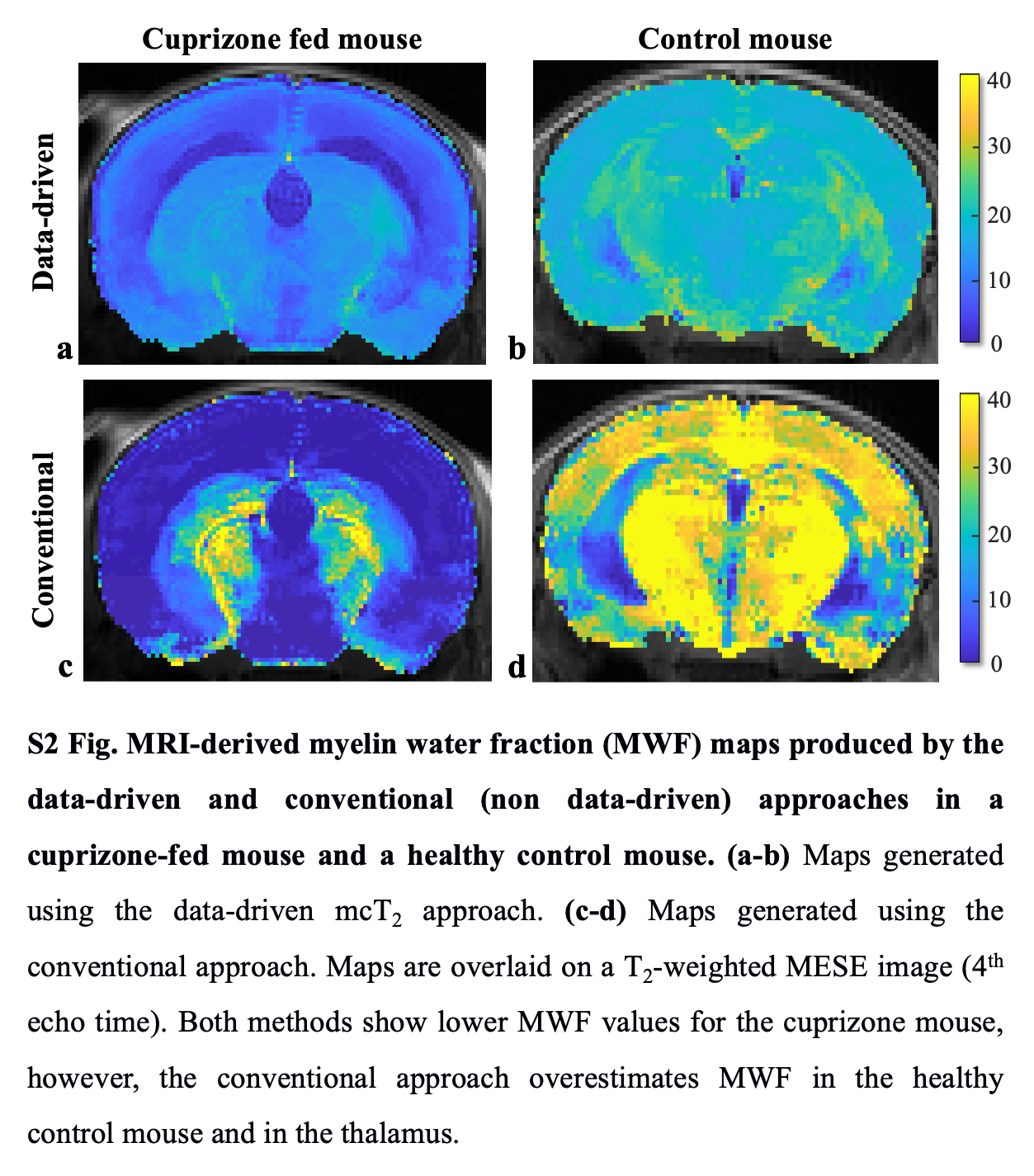

Supplement: S2 Fig — (a-b) Maps generated using the data-driven mcT2 approach. (c-d) Maps generated using the conventional approach. Maps are overlaid on a T2-weighted MESE image (4th echo time). Both methods show lower MWF values for the cuprizone mouse, however, the conventional approach overestimates MWF in the healthy control mouse and in the thalamus. (TIFF) [file pone.0323614.s002.tiff]

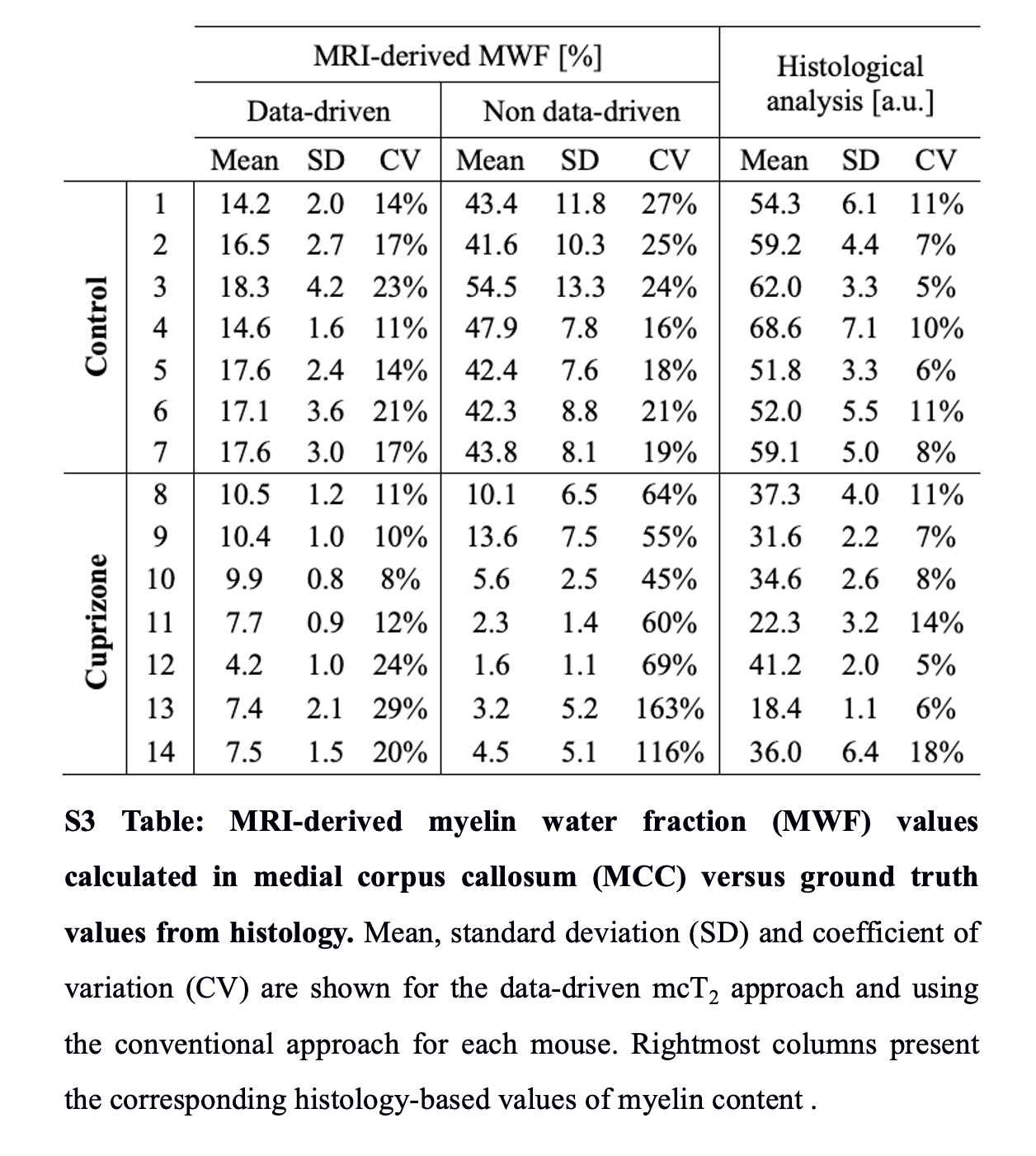

Supplement: S1 Table — Mean, standard deviation (SD) and coefficient of variation (CV) are shown for the data-driven mcT2 approach and using the conventional approach for each mouse. Rightmost columns present the corresponding histology-based values of myelin content. (TIFF) [file pone.0323614.s003.tiff]
